# When it starts in the skin and goes to the lungs, where does it stop?

**DOI:** 10.1002/rcr2.1043

**Published:** 2022-09-27

**Authors:** Ana Rita Catarino Ferro, Ana Margarida Ferreira Campos

**Affiliations:** ^1^ Pulmonology Department Centro Hospitalar Tondela‐Viseu Viseu Portugal

**Keywords:** Birt‐Hogg‐Dubé syndrome, fibrofolliculomas, pneumothorax, renal cancer

## Abstract

This clinical case reports a rare disease—Birt‐Hogg‐Dubé Syndrome—characterized by skin lesions and multiple lung cysts. Because of its rarity, BHDS is likely undiagnosed and mistaken for primary spontaneous pneumothorax or emphysema. An early diagnosis is important to set up screening for renal cancer in patients and affected relatives.

## Graphical abstract

This case reports a patient diagnosed with Birt‐Hogg‐Dubé syndrome (BHDS), a rare disease, with an increased risk of fibrofolliculomas in the skin, multiple lung cysts predisposing to recurrent pneumothorax and increased risk of renal cancer. Its clinical expression is variable, which makes the diagnosis and management difficult.

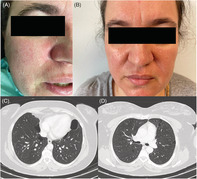

## CLINICAL IMAGE

**FIGURE 1:**
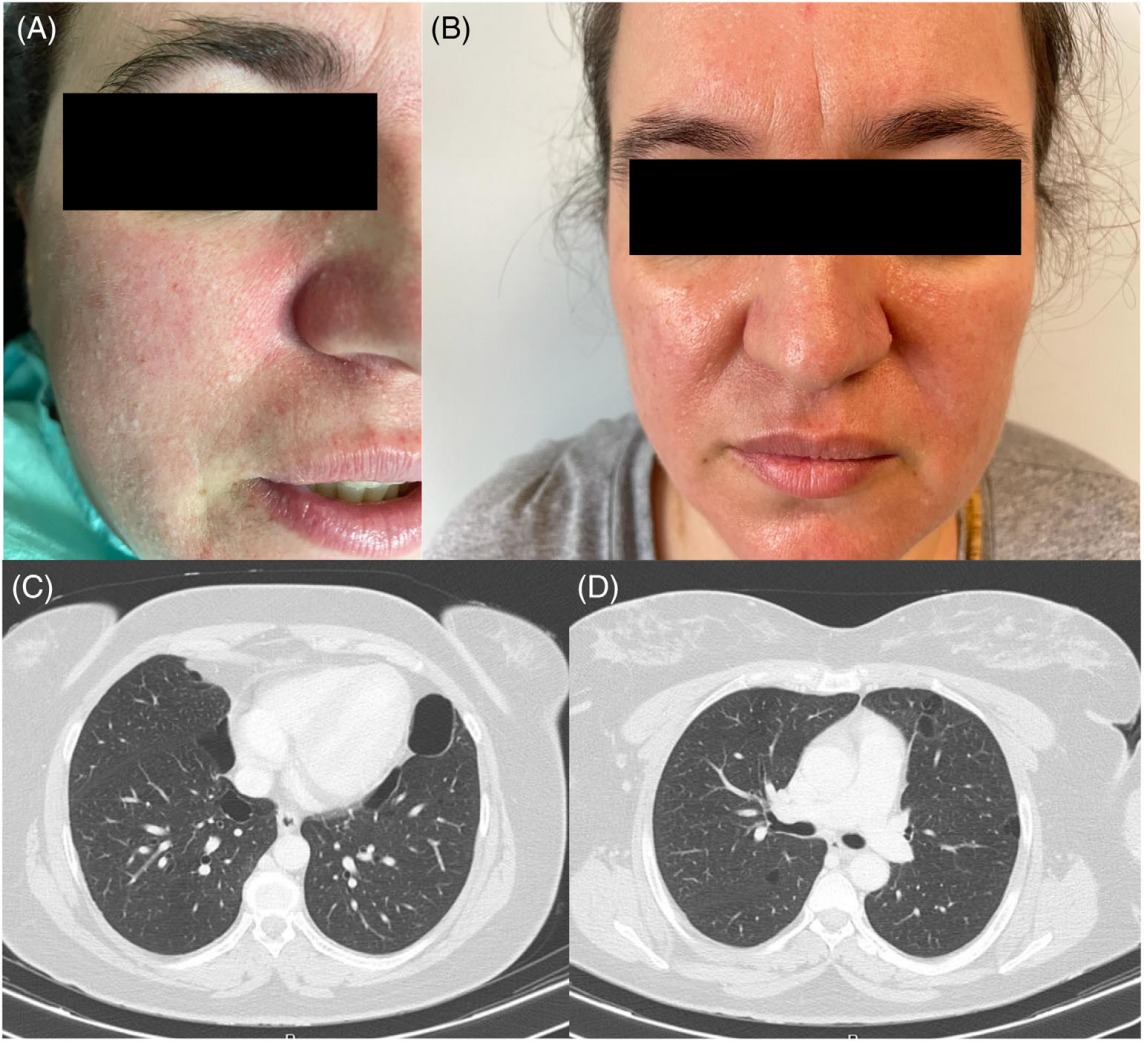
(A, B) Multiple whitish dome‐shaped papules visible in the nose and paranasal area. Biopsy demonstrated fibrofolliculomas. (C, D) Chest computed tomography scan axial cut showed thin walled, round and ovoid pulmonary cysts predominanting in the lower‐medial zone of both lungs

A previously healthy 41‐year‐old patient, referred to the Pulmonology Department for fibrofolliculomas and trichodiscomas in the face (Figure 1A, B) and changes in chest computed tomography (CT). She had no respiratory or constitutional symptoms. The physical examination was unremarkable. Laboratory data, including alfa‐1 antitrypsin, were normal. Chest CT showed multiple cysts in the basal parts of the lungs (Figure 1C, D). Pulmonary function tests and magnetic resonance imaging of the kidneys were normal. Genetic screening revealed that the patient had a mutation in the *FLCN* gene, c.1015C>T, which confirmed the diagnosis of Birt‐Hogg‐Dubé Syndrome (BHDS). Family members were offered genetic counselling and investigations. The patient received CO_2_ laser treatment and was informed about the risk of spontaneous pneumothorax.

BHDS is a rare disease with an increased risk of fibrofolliculomas in the skin, multiple lung cysts predisposing to recurrent pneumothorax and increased risk of renal cancer.^1^, ^2^ Its clinical expression is variable, which makes the diagnosis and management difficult.^1^

## AUTHOR CONTRIBUTIONS

Ana Rita Catarino Ferro did the conception and design of the work. All authors revised critically the work.

## CONFLICT OF INTEREST

None declared.

## ETHICS STATEMENT

The authors declare that appropriate written informed consent was obtained for the publication of this manuscript and accompanying images.

## Data Availability

The data that support the findings of this work are available on request from the corresponding author.
